# Differential Somatic Cell Count as a Novel Indicator of Milk Quality in Dairy Cows

**DOI:** 10.3390/ani10050753

**Published:** 2020-04-26

**Authors:** Giorgia Stocco, Andrea Summer, Claudio Cipolat-Gotet, Lucio Zanini, Diego Vairani, Christos Dadousis, Alfonso Zecconi

**Affiliations:** 1 Department of Veterinary Science, University of Parma, Via del Taglio 10, 43126 Parma, Italy; giorgia.stocco@unipr.it (G.S.); andrea.summer@unipr.it (A.S.); christos.dadousis@unipr.it (C.D.); 2 Associazione Regionale Allevatori Lombardia, Via Kennedy 30, 26013 Crema, Italy; l.zanini@aral.lom.it (L.Z.); d.vairani@aral.lom.it (D.V.); 3 Department of Biomedical, Surgical and Dental Sciences, University of Milano, Via Celoria 10, 20133 Milano, Italy; alfonso.zecconi@unimi.it

**Keywords:** differential somatic cell count, somatic cells, milk quality

## Abstract

**Simple Summary:**

Recently, high-throughput instruments have been used to analyze milk differential somatic cell count, represented by the combined proportion of polymorphonuclear leukocytes and lymphocytes, providing indirect information on the udder inflammation status of dairy cows. No information is available about the relationship between differential somatic cell count and milk quality, so the aim of this study was to investigate the effect of differential somatic cell count on the composition of a large number of individual milk samples. Results showed that milk quality worsened when differential somatic cell count was high. In particular, it was evidenced lower milk fat, protein, casein contents and casein index, and augmented milk fatty acids could be found with an increasing differential somatic cell count level. These findings confirmed that differential somatic cell count could be a new informative tool for dairy farmers to monitor the quality of milk.

**Abstract:**

Recent available instruments allow to record the number of differential somatic cell count (DSCC), representing the combined proportion of polymorphonuclear leukocytes and lymphocytes, on a large number of milk samples. Milk DSCC provides indirect information on the udder health status of dairy cows. However, literature is limited regarding the effect of DSCC on milk composition at the individual cow level, as well as its relation to the somatic cell score (SCS). Hence, the aims of this study were to (i) investigate the effect of different levels of DSCC on milk composition (fat, protein, casein, casein index, and lactose) and (ii) explore the combined effect of DSCC and SCS on these traits. Statistical models included the fixed effects of days in milk, parity, SCS, DSCC and the interaction between SCS × DSCC, and the random effects of herd, animal within parity, and repeated measurements within cow. Results evidenced a decrease of milk fat and an increase in milk fatty acids at increasing DSCC levels, while protein, casein and their proportion showed their lowest values at the highest DSCC. A positive association was found between DSCC and lactose. The interaction between SCS and DSCC was important for lactose and casein index, as they varied differently upon high and low SCS and according to DSCC levels.

## 1. Introduction

Somatic cell count (SCC) is used worldwide as an indicator of udder health in dairy animals and to indirectly monitor the quality of milk [1]. In dairy cows, high SCC is an indicator of subclinical mastitis [1,2], low milk quality [3,4], poor ability of the milk to coagulate [5,6], and reduced cheese yield and recovery of milk nutrients in the curd [7,8,9]. However, although SCC is a valid quantitative trait for the identification of the inflammation status of the mammary gland, it does not provide information on the distribution of each cell population. This information, however, could be extracted by the variation of polymorphonuclear leukocytes (PMN), lymphocytes, and macrophages, which reflects the extent of the inflammatory response of the animal [10]. In fact, the modifications in the amount of these different cell populations in milk according to the udder health conditions relates to the actual inflammatory status of the mammary glands [11,12]. Moreover, there is evidence that different proportions of these cell populations, within specific SCC levels, could modify the final characteristic of dairy products [13,14], as well as different categories of environmental microorganisms (i.e., Clostridia) that, although nonpathogenic, have a strong unfavorable dairy activity [15].

Despite this, due to the absence of a fast and high-throughput milk analyzer, able to count differential cell populations in milk, this information was unavailable [16]. Recent technological advances have changed this situation and new tools have become available that count milk differential somatic cells (DSCC, defined as PMN + lymphocytes and expressed in percentage) and can be integrated in the daily recording system of individual milk samples [16,17]. Previous studies investigated the variation of DSCC in milk before, during, and after induced inflammation in dairy cows [18,19]. Moreover, DSCC has been proposed as a useful biomarker in milk to indirectly identify mastitis, revealing inflammatory reactions also at low SCC [20], and being able to determine the stage of mastitis in combination with SCC [19]. Genetic aspects of DSCC have also been preliminarily investigated in Holstein cows [21], suggesting the consideration of DSCC in breeding programs for the enhancement of bovine resistance to mastitis.

However, as abovementioned, despite the extended literature on the relation between SCC and milk quality and productive performances of dairy animals [2,4], few studies investigated the relationship between the different cell populations and milk composition. Moreover, these studies were mainly focused on the PMN cell type [22,23], or on the contribution of PMN [14] and macrophages [13] in the proteolysis in bulk milk samples, and not in individual milk samples. Thus, there is no information on the relationships between DSCC and milk composition at the animal level. Given the growing interest in DSCC in the dairy sector, it would be of importance to explore the relationship between different DSCC levels and milk composition changes. This new knowledge could be further used by dairy farmers to better monitor the quality of their milk and, eventually, to improve the management practices at farm level, as well as for breeding purposes at a population level. For these reasons, the aims of this study were to (i) investigate the effect of different levels of DSCC on milk composition (fat, protein, casein, casein index, lactose) of individual dairy cows, and (ii) explore the combined effect of DSCC and SCS on these quality traits.

## 2. Materials and Methods 

### 2.1. Ethical Statement

All the dairy cows involved in this study were reared in commercial private farms and were not subjected to any invasive procedures. Milk samples used for the analyses were collected by technicians from the Breeders’ Association of Lombardia Province (Italy), and were hence certified by the local authority.

### 2.2. Milk Sample Collection and Analyses

A total of 440 lactating cows reared in 4 herds located in Lombardy region (North-West of Italy) were repeatedly sampled for milk quality analyses during morning milking, from September to December 2018. Briefly, the 4 herds were characterized by 165, 86, 102, and 87 cows in milk, with an average milk production of 34.8, 33.5, 29.5, and 30.1 kg/d, respectively. Herds were selected in order to be representative in terms of size and management with respect to the farms located in the Lombardy region, and characteristics of sampled farms have been previously reported in Zecconi et al. [17]. Different milking parlors were present: rotary, automatic, parallel and herringbone. Milk produced from these herds was intended for cheese-making (2 herds), organic production (1 herd), and as fluid product (1 herd). Feeding consisted in total mix ration (3 herds), and free forage and automatic feeding concentration for the herd with organic production. Twelve cows were not included in the dataset because the number of observations per cow was lower than 3. So, 428 cows (3–15 observations per cow, for a total of 4745 records) were considered for the statistical analysis. Cows belonged to Holstein-Friesian, Brown Swiss, and Simmental breeds. Milk samples (without preservative) were placed at 4 °C immediately after collection and analyzed within 24 h from the sampling by CombiFoss 7 (Foss Electric A/S, Hillerød, Denmark). Milk fat, protein, casein, lactose, and groups of fatty acids (FA; SFA = saturated; UFA = unsaturated; MUFA = monounsaturated; and PUFA = polyunsaturated) were measured with a Fourier-transform infrared spectrophotometer (FT-IR) MilkoScan 7 (calibrated according to ISO 9622/IDF 141:2013), while SCC and DSCC (PMN + lymphocytes, %) were determined with Fossomatic 7DC (according to ISO 13366-2/IDF 148-2:2006 standards). SCC was transformed into the logarithmic [log_2_(SCC × 10^−5^) + 3] somatic cell score (SCS) [24]. Casein index was calculated as the casein to protein ratio, and multiplied by 100. 

### 2.3. Statistical Analysis

An ANOVA analysis (MIXED procedure; SAS Institute Inc., Cary, NC, USA) was used to assess the association of DSCC with various milk quality traits, including DSCC as a fixed effect. Our dataset includes repeated measurements of milk composition within a cow and these are correlated to each other, especially for two subsequent measures. It is important to incorporate this feature into the model, specifying a correlation structure among the repeated measurements. Therefore, an autoregressive (Order 1) structure that considers the correlations between subsequent repeated milk measurements, was fitted. Moreover, following Zecconi et al. [17], a threshold of 68.5% for DSCC was applied and, upon this, 4 classes were created. This threshold was identified by using receiver operating characteristic (ROC) calculation curves and adopting a threshold of 200,000 cells/ml as a gold standard for SCC [25,26,27]. The 4 classes were created based on 5% or 10% deviance from the aforementioned threshold for the two middle classes, while the two extreme classes included the tails of the distribution. More precisely, in the first model (M-5_DSCC_), the DSCC classes were: class 1: <63.5%; class 2: 63.5%–68.5%; class 3: 68.5%–73.5%; and class 4: >73.5%. The second model (M-10_DSCC_), was less stringent, at a 10% deviance relative to the 68.5% threshold (class 1: <58.5%; class 2: 58.5%–68.5%; class 3: 68.5%–78.5% and class 4: >78.5%). Ranges were chosen in order to have a sufficient sample size within each class of DSCC, and to distinguish relevant modifications in milk composition across DSCC classes. Frequencies for each class of DSCC for both M-5_DSCC_ and M-10_DSCC_ of milk samples collected during the experimental trial among herds, together with mean values of days in milk, parity, and daily milk yield for individual cows are reported in Table 1.

The statistical model, for both M-5_DSCC_ and M-10_DSCC_, was the following:

y_mnopqrst_ = μ + Herd_m_ + DIM_n_ + Parity_o_ + Animal_p_ (Parity_o_) + Rep_q_ + SCS_r_ + DSCC_s_ + SCS_r_ × DSCC_s_ + e_mnopqrst_

where y_mnopqrst_ is the observed trait (fat, protein, casein, casein index, lactose, SFA, UFA, MUFA, and PUFA); μ is the overall intercept; Herd_m_ is the random effect of the m^th^ herd (m = 1 to 4) ~$N\left(0,I\sigma_{h}^{2}\right)$; DIM_n_ is the fixed effect of the n^th^ class of days in milk [*n* = 1 to 5; class 1: 5–65 d (1,117 samples); class 2: 66–125 d (970 samples); class 3: 126–185 d (613 samples); class 4: 186–240 d (548 samples); class 5: >240 d (829 samples)]; Parity_o_ is the fixed effect of the o^th^ class of parity [o = 1 to 4; class 1: 1st parity (1621 samples); class 2: 2nd parity (1542 samples); class 3: 3rd parity (807 samples); class 4: ≥4th parity (775 samples)]; Animal_p_ is the random effect of the pth animal (p = 1 to 428) within the oth class of Parity~$N\left(0,I\sigma_{a}^{2}\right)$; Rep_q_ is the random effect accounting for environmental covariances among records within cows~$N\left(0,H\sigma_{r}^{2}\right)$; SCS_q_ is the fixed effect of the qth class of SCS [q = 1 to 2; class 1: <5.00 (2956 samples); class 2: ≥5.00 (1789 samples)]; DSCC_r_ is the fixed effect of the rth class of DSCC (as aforementioned, classes were built upon the 68.5% DSCC threshold); SCS_q_ × DSCC_r_ is the fixed effect of the interaction between the qth class of SCS and the rth class of DSCC; e_mnopqrst_ is the random residual ~$N\left(0,I\sigma_{e}^{2}\right)$; I is an identity matrix, H is an auto regression of order 1 correlation matrix of the environmental correlations between pairs of records on the same animal, $\sigma_{h}^{2}$, $\sigma_{a}^{2}$, $\sigma_{r}^{2}$ and $\sigma_{e}^{2}$ are the herd, animal, permanent environmental among repeated records, and residual variances, respectively. Orthogonal contrasts were estimated between least square means (LS Means) of milk traits for the DSCC effect for both M-5_DSCC_ and M-10_DSCC_ models:

(a) milk samples close (below + above, classes 2 + 3) to the threshold vs. class 1 of DSCC (lowest values); 

(b) milk samples with DSCC values close the threshold (class 2 vs. class 3);

(c) milk samples close (below + above, classes 2 + 3) to the threshold vs. 4 class of DSCC (highest values).

## 3. Results and Discussion

Descriptive statistics of milk components and FA of individual milk samples are reported in Table 2. Many traits exhibited high variability (CV, %), possibly due to the individual characteristics of the animals (especially for those milk components which phenotypic variation is linked to the genetics, as protein and casein contents) and to the differences among farms (i.e., milk fat). The mean value of SCS was 4.91 (corresponding to ~375,000 cells/mL of milk) and ranged from 4.08 (5th percentile) to 6.07 (95th percentile), corresponding to SCC at roughly 200,000 and 800,000 cells/mL, respectively. The average of DSCC was 57.8%, ranging from 29.8% to 84.2%. The proportion of macrophages can be calculated by subtracting DSCC from 100%, so that macrophages in this study were on average 42.2%. The number of cells available to determine DSCC clearly depends on SCC, and to obtain a sufficient measurement of accuracy, the performance range for the method was defined to be between 50,000 and 1,500,000 cells/mL [16], so the DSCC and SCC values included in this study were highly reliable.

### 3.1. Effect of Somatic and Differential Somatic Cells Count

Results from the analysis of variance for milk components and groups of FA are presented in Table 3. The *F*- and *p* for the herd, animal, repeated measurements, DIM and parity were almost identical between model M-5_DSCC_ and M-10_DSCC_, hence only the values of the M-5_DSCC_ model are presented in the Table 3. Since those effects were not within the aims of the study, they were not discussed further in the text.

The effect of SCS (two classes: <5.00 and ≥5.00, corresponding to <400,000 and ≥400,000 cells/mL, respectively) in both models M-5_DSCC_ and M-10_DSCC_ resulted significant (excluding casein index and UFA group) with nearly the same extent for all the traits considered (Table 3). Milk samples with ≥5.00 SCS were characterized by lower values of lactose (4.80% vs. 4.89%), SFA (68.2 vs. 68.6 g/100 g total FA) and PUFA (4.60 vs. 4.72 g/100 g total FA), and higher values of fat (3.74 vs. 4.19%), protein (3.53% vs. 3.47%), casein (2.77% vs. 2.72%), and MUFA (27.3% vs. 26.7 g/100 g total FA) contents, compared to milk samples with <5.00 SCS (LS Means from M-5_DSCC_; data not shown). These findings are in agreement with previous studies focusing on the effect of SCC on milk composition [23,28]. 

In the literature, few studies considered the different somatic cell populations. Moreover, those studies were mainly focused on PMN cell type. Therefore, a straightforward comparison with our results was not possible. In the present study, the effect of DSCC was significant both for milk composition and groups of FA (Table 3). Outcomes obtained from the different DSCC ranges are further discussed in the following paragraphs. 

Milk with low DSCC, often associated with healthy animals, is characterized by a relative high number of macrophages and lymphocytes and low PMN, while the opposite is expected for a high number of PMN [11,16,29]. Previous studies investigating the relationships between PMN and quality traits in bovine bulk milk samples, evidenced that high number of PMN cells was associated with reduced fat [23] and increased lipolysis rate as a consequence of the release of lipolytic enzymes [30,31]. Hence, in our case, it is reasonable to suppose that the diminution of fat in milk samples with high DSCC could be due to the specific rise of PMN population. 

Although DSCC represents the combined proportion of PMN and lymphocytes, it is acknowledged that it reflects the opposite association between PMN and macrophages, while the proportion of lymphocytes appears to be quite constant [16].

**Figure 1:**
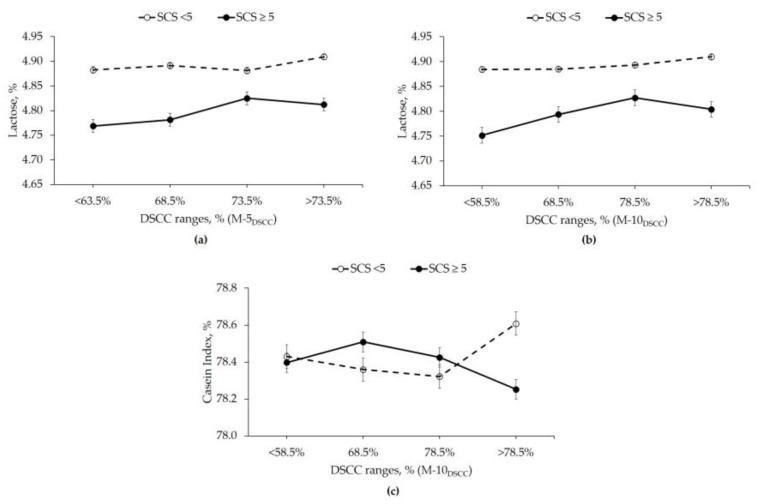
Effect of interaction of SCS×DSCC according to M-5_DSCC_ model on lactose (**a**) (*p* < 0.01), and SCS × DSCC according to M-10_DSCC_ model on lactose (**b**) (*p* < 0.001) and casein index (**c**) (*p* < 0.05).

Findings from the interaction SCS × DSCC are important in explaining the change of each milk component linked to DSCC concentration, and not just to SCC. This was significant for lactose in the M-5_DSCC_, and also for the casein index when M-10_DSCC_ was tested (Table 3). From Figure 1a, it is evident that milk samples with <5.00 SCS contained higher lactose content compared to samples with ≥5.00 SCS, with a slight increase of this component in milk samples with the highest DSCC content. Then, in milk samples with ≥5.00 SCS, lactose tended to increase across DSCC, till 73.5%, and finally it slightly decreased. These trends were better noticeable in the M-10_DSCC_ (Figure 1b). Hence, it could be hypothesized that lactose was able to maintain the osmotic equilibrium in milk across increasing levels of DSCC for cows with SCS <5.00. On the contrary, in those cows with SCS ≥5.00, increased level of lactose was observed from the very beginning, possibly due to the augmented glucose demand [32,33] as a consequence of the first responses of the cow toward inflammation (suggested by the increasing DSCC values induced by the increase of PMN proportion), but again it decreased in the last DSCC range, suggesting the impaired lactose synthesis. 

Regarding the casein index (Figure 1c), its gradual decrease in milk samples with <5.00 SCS till 78.5% DSCC, and then the sudden increase over 78.5% could be explained by the proteolytic enzymes released by the changing proportion of cell populations within DSCC, and also considering the remaining cell population out of DSCC that is represented by macrophages. 

Polymorphonuclear leukocytes have different enzyme profiles with different activity of degradation of proteins and caseins with respect to macrophages. In particular, the extent of the activity of degradation changes according to the quantity of a certain cell population (for example, PMN contain very active proteases, but macrophages engulfing pathogens produce 5–6 times more proteases than neutrophils) [34]. The pattern of casein index showed an opposite trend in milk samples with ≥5.00 SCS compared to milk samples with <5.00 SCS. Indeed, during an inflammation status, the components synthesized in the gland (αs-casein, β-casein, β-lactoglobulin, and α-lactalbumin) decrease, while those components coming from the blood (mainly immunoglobulins and serum albumin) increase [35]. These changes in the passage of components from blood into the milk seem clearly represented by Figure 1c. This result is important especially for the milk destined to cheese-making, as low casein index is associated with worsened ability to coagulate [36]. 

### 3.2. Milk Samples Close to the DSCC Threshold (Below + Above) vs. Low DSCC

The LS Means, accompanied with the *F*-value and significance of their orthogonal contrasts, for DSCC ranges according to M-5_DSCC_ and M-10_DSCC_ are summarized in Table 4 and Table 5 respectively. As evidenced from the first contrast in Table 4, milk samples close to the threshold (class 2 and 3; M-5_DSCC_) showed an inferior value of fat compared to milk samples with class 1 of DSCC (lowest DSCC), while the opposite was evidenced for lactose content. These effects were much more statistically marked moving from M-5_DSCC_ to the corresponding first contrast of M-10_DSCC_ (Table 5). As previously mentioned, PMN is the predominant cell population within somatic cells once inflammation occurs, being rapidly recruited at the beginning of an acute inflammatory response [10], and lipolysis rate is increased in milk from infected animals, as a consequence of lipolytic enzymes released by PMN rather than macrophages and lymphocytes [30]. These secreted lipolytic enzymes bind to the fat globule membranes in milk and expose fat to degradation. Results about the effect of DSCC on lactose of individual milk samples are not available in the literature, although Wickström et al. [23] did not find any effect of PMN on lactose content of bulk milk samples. Besides the differences in the number of samples and the type of milk used (individual or bulk) between studies, in our case the DSCC ranges were built upon a threshold previously chosen for the same dataset to identify subclinical mastitis in dairy cows with an accuracy of 0.82, and a sensitivity and specificity of 0.76 and 0.81, respectively [17]. Hence, at the beginning of the inflammation status (in our case it could be supposed to be represented by the milk samples around the threshold; classes 2 and 3), the increased demand for glucose in the inflammatory site previously hypothesized, and the possible enhanced activation of β1,4-galactosyltranferase in response to inflammation [37] could have led to a higher synthesis of lactose [38]. With respect to this novel finding, it is important to remind that, although the species differ greatly from bovine, studies effectuated in vitro in humans [39] and in vivo on mice [40], evidenced that lactose exerts strong modulatory effects on the immune responses, in particular those mediated by neutrophils and macrophages. In this regard, genome-wide association studies could be used as a powerful technique to investigate the genetic pathways [41] that activate and regulate the different levels of DSCC in milk. These results about the effect of different ranges of DSCC on milk composition are particularly important in the case of milk destined for cheese-making, as the concentration of each component in milk strongly influences coagulation properties and the cheese-making ability of milk [42].

### 3.3. Milk Samples Close to the DSCC Threshold (Below vs. Above)

Considering now the results obtained from the second contrast, comparing the two classes below vs. above the threshold (class 2 vs. class 3), if in the case of the M-5_DSCC_ the difference was significant for UFA and PUFA (higher in milk samples in class 3; Table 4), in M-10_DSCC_ the effect of DSCC was significant for fat, lactose and PUFA (Table 5). This indicates that, for cows in a ±10% range from the threshold, DSCC could be a useful indirect indicator of changes in milk composition, while in the case of M-5_DSCC_, DSCC had less efficacy. For example, lower fat content was observed in milk samples in class 3 compared to those in class 2 (3.88% vs. 4.05%, respectively for class 3 and 2 in M-10_DSCC_), again attributable to the lipolysis effect from enzymes released by PMN cells [30]. On the contrary, higher lactose content was observed in milk samples belonging to class 3 compared to those in class 2 (4.86% vs. 4.84%, respectively for class 3 and 2 in M-10_DSCC_), suggesting that this trend must be explained by the complex open self-regulatory system of the mammary gland, also supposing that lactose might have a role in the modulation of the immune response, that cannot be detected by only using the association with SCS. To the best of our knowledge, no study has investigated the effect of DSCC or different population cells on milk fatty acids. However, some dated results are available from the literature as regards to the effect of mastitis on individual FA. For example, Randolph and Erwin [43] evidenced that milk samples positive for mastitis showed higher concentrations of FA compared to negative ones, in particular some short- (butyric, caproic, caprylic, and capric) and long-chain acids (palmitic, palmitoleic, oleic, and linoleic). Those authors also showed that milk from infected cows had lower fat content, higher lipase activity and acid degree values compared to milk from healthy cows, that explain the lower FA content in mastitic milk. As regards to PUFA, it has been observed a higher content in milk samples belonging to class 3 compared to those in class 2 (4.69 vs. 4.62 g/100 g total FA, respectively for class 3 and 2 in M-10DSCC). An interesting explanation of why only some groups of FA significantly increased just after the threshold (UFA and PUFA in the case of M-5_DSCC_, PUFA in the case of M-10_DSCC_) could be provided by specific studies on the effects of FA on the oxidative burst and viability of bovine neutrophils in vitro, where it was demonstrated that fatty acids (a mixture composed of palmitic, palmitoleic, stearic, oleic, and linoleic acids was used) are endowed with immunomodulatory effects [44]. Rezamand and McGuire [45] evidenced a clear contribution of trans fatty acids (elaidic and linoleidic acid) during inflammation in bovine mammary epithelial cells. Essential fatty acids, notably linoleic and α-linolenic acids, the main contributors to PUFA [46], have direct effects on physiological processes such as cellular membrane integrity, hormonal pathways, and immune function [47]. However, given these results, and due to the complexity of the biological pathways involved in FA synthesis, we can only speculate on these alternative explanations and we have to interpret with caution the differences groups of FA (i.e., FT-IR measurements, low significance of the contrasts).

### 3.4. Milk Samples Close to the DSCC Threshold (Below + Above) vs. High DSCC 

Considering the results obtained by comparing milk samples close to the threshold (below + above; class 2 + 3) with milk samples in class 4 (the highest DSCC level), it is evident that most of milk components reached their lowest values in milk samples with high DSCC (> 73.5% in the case of M-5_DSCC_ and > 78.5% in the case of M-10_DSCC_; Table 4 and Table 5). In particular, with M-5_DSCC_ the changes in milk composition in the highest DSCC (>73.5%, class 4) were linked to the fat (reaching its lowest value) and lactose (reaching its highest value) contents, and MUFA, whose content in class 4 of DSCC was higher compared to the average value of the two classes around the threshold. In the case of M-10_DSCC_ milk modifications regarded fat, protein and casein, that reached their lowest values in the last DSCC class (class 4; Table 5). Findings from this last contrast confirmed that high DSCC (>73.5% in the case of M-5_DSCC_ and >78.5% in the case of M-10_DSCC_; Table 4 and Table 5) may cause direct adverse effects on milk quality, such as fat reduction and decreased protein and casein contents. These modifications in milk composition, mainly attributable to the enzymatic reactions mediated by PMN and stimulation of plasmin activity [48], evidence that the shelf life of liquid milk could be potentially shortened, and coagulation ability could be compromised. Certainly, these aspects need to be further investigated. However, it is recognized that small variations in milk protein concentration are sufficient to exert quite a large effect on coagulation properties [49]. In addition, low milk fat and casein are associated with a worsened coagulation ability of milk [50] and with reduced cheese yield and recovery of nutrients in the curd [51].

## 4. Conclusions

To the best of our knowledge, this is the first study investigating the effect of DSCC on milk composition traits from individual milk samples. The importance of gaining knowledge from DSCC, rather than just the overall SCC, in relation to changes in milk composition was evidenced. Results showed that modifications in milk composition are associated with different levels of DSCC. In particular, the possibility to divide DSCC into different classes built upon a threshold defined to identify mastitis allowed to distinguish the specific interval in which milk composition changes. Fat and lactose were the milk components affected at most by the early DSCC rises, and across DSCC levels. Protein and casein decreased significantly at very high DSCC level (>78.5%), while casein index was affected when DSCC was combined with low or high SCS. Also, a positive association between DSCC and lactose was observed. For milk samples close to the DSCC threshold (68.5%), DSCC could be used as a useful indirect indicator of changes in milk composition in a ±10% range from the threshold (M-10_DSCC_). The importance of the interaction between SCS and DSCC was highlighted, because the trends of lactose and casein index varied upon high and low SCS and according to DSCC classes. In this way, DSCC (alone and in combination with SCC) could be used to monitor the quality of milk along the dairy chain, from the farm (i.e., by reducing the use of antimicrobials) to the dairy plant (i.e., by including DSCC to further assess the characteristics and the economic value of milk). Moreover, the novel information provided by this study paves the way for new research insights emerging from the investigation of the relationships between different levels of DSCC and SCS, and coagulation traits, cheese-making ability of milk, and cheese quality characteristics.

## Figures and Tables

**Table 1:** Mean values ^1^ of days in milk, parity and daily milk yield for individual cows and frequencies for each class of Differential Somatic Cell Count (DSCC) of M-5_DSCC_ and M-10_DSCC_ of milk samples collected during the experimental trial among herds.

Item	Herd 1	Herd 2	Herd 3	Herd 4
Days in milk, d	131	184	170	158
Parity	2.39	2.43	2.07	2.08
Milk Yield, kg/d	34.8	33.5	29.5	30.1
M-5_DSCC_ ranges ^2^				
<58.5% (class 1)	51.0	48.6	75.2	54.7
58.5–68.5% (class 2)	20.5	10.6	5.6	10.0
68.6–78.5% (class 3)	16.1	12.3	7.3	10.3
>78.5% (class 4)	12.4	28.6	11.9	25.1
M-10_DSCC_ ranges ^3^				
<63.5% (class 1)	62.2	39.4	67.3	45.5
63.5–68.5% (class 2)	9.3	19.8	13.5	19.2
68.6–73.5% (class 3)	8.6	25.3	12.3	19.2
>73.5% (class 4)	19.9	15.6	6.9	16.2

^1^ Mean values for days in milk and parity among herds were estimated from the mean values for each cow. Mean values of daily milk yield among herds were estimated from the first milk sampling for each cow; ^2^ M-5_DSCC_ = class 2 and 3 were created with ±5% deviance from the 68.5% DSCC threshold; ^3^ M-10_DSCC_ = class 2 and 3 were created with ±10% deviance from the 68.5% DSCC threshold.

**Table 2:** Descriptive statistics of milk components and groups of fatty acids (FA) of individual milk samples.

Trait	Mean	CV ^1^, %	Percentile ^2^		Skewness	Kurtosis
			P5th	P95th		
Milk components						
Fat, %	4.10	23	2.50	5.62	−0.02	0.32
Protein, %	3.55	11	2.93	4.26	0.25	−0.29
Casein, %	2.79	12	2.27	3.38	0.24	−0.31
Casein index ^3^, %	78.5	2	76.2	80.6	−0.19	0.18
Lactose, %	4.87	4	4.51	5.16	−0.49	0.15
SCS ^4^	4.91	12	4.08	6.07	0.73	0.50
DSCC ^5^, %	57.75	29	29.8	84.2	−0.11	-0.81
Milk FA ^6^, g/100 g total FA						
SFA	68.7	6	60.7	75.0	−0.48	−0.23
UFA	30.8	15	24.0	39.0	0.37	−0.13
MUFA	26.8	15	21.2	34.1	0.59	0.21
PUFA	4.49	16	3.46	5.88	0.54	0.19

^1^ CV, % = coefficient of variation; ^2^ Percentile = 5th and 95th percentiles, which indicate the upper and lower 5% limits in the 2-tailed distribution of data; ^3^ Casein index = casein to protein ratio, multiplied by 100; ^4^ SCS = Somatic Cell Score; ^5^ DSCC = Differential Somatic Cell Count; ^6^ FA= Fatty Acids; SFA = Saturated; UFA = Unsaturated; MUFA = Monounsaturated; PUFA = Polyunsaturated.

**Table 3:** Analysis of variance of M-5_DSCC_ and M-10_DSCC_ models ^1^ for milk components and groups of fatty acids (FA), with *F*-value and significance for fixed effects (DIM, parity, SCS, DSCC, and SCS × DSCC) and the proportion of variance (in root mean square) explained by random effects (herd, animal within parity, and repeated measurements within cow) and by the residual (RMSE).

Trait	Random Effects ^2^			RMSE ^3^	Fixed Effects (*F*-Value and Significance)							
	Herd	Animal	Rep. Measurement		DIM ^4^	Parity	SCS ^5^		DSCC ^6^		SCS × DSCC	
							M-5_DSCC_	M-10_DSCC_	M-5_DSCC_	M-10_DSCC_	M-5_DSCC_	M-10_DSCC_
Milk components, %												
Fat	0.37	0.55	0.32	0.68	17.6 ***	1.4	120.1 ***	93.0 ***	12.9 ***	10.7 ***	0.5	1.3
Protein	0.17	0.25	0.66	0.20	88.0 ***	1.7	28.7 ***	28.1 ***	1.8	3.4 *	1.5	0.0
Casein	0.14	0.22	0.63	0.16	120.2 ***	2.7 *	32.8 ***	29.8 ***	1.7	2.8 *	1.7	0.1
Casein index ^7^	0.25	0.79	0.42	0.91	123.4 ***	11.5 ***	0.5	0.3	0.5	0.4	2.2	3.2 *
Lactose	0.00	0.12	0.52	0.11	17.4 ***	22.4 ***	205.5 ***	187.9 ***	8.7***	12.4 ***	5.2 **	8.7 ***
Milk FA ^8^, g/100 g total FA												
SFA	3.30	2.18	0.66	2.76	47.1 ***	6.1 ***	6.5 *	8.5 ***	1.8	1.1	1.2	2.3
UFA	3.01	2.17	0.46	3.12	41.6 ***	6.4 ***	0.9	1.3	2.8 *	2.3	0.8	1.1
MUFA	2.70	1.95	0.66	2.60	46.0 ***	6.4 ***	13.1 ***	13.7 ***	2.3	1.1	1.5	2.0
PUFA	0.64	0.38	0.46	0.41	42.7 ***	0.2	21.2 ***	11.6 ***	2.7 *	2.2	0.1	1.2

^1^ Model M-5_DSCC_ = DSCC classes, %: class 1, <63.5; class 2, 63.5–68.5; class 3, 68.5–73.5; class 4, >73.5; model M-10_DSCC_ = DSCC classes, %: class 1, < 58.5; class 2, 58.5–68.5; class 3, 68.5–78.5; class 4, >78.5; ^2^ Random effects of herd, animal and repeated measurements expressed as root mean square; ^3^ RMSE = Root Mean Square Error; ^4^ DIM = Days in milk; ^5^ SCS = Somatic Cell Score; ^6^ DSCC = Differential Somatic Cell Count; ^7^ Casein index = casein to protein ratio, multiplied by 100; ^8^ FA = Fatty Acids; SFA = Saturated; UFA = Unsaturated; MUFA = Monounsaturated; PUFA = Polyunsaturated; * *p* < 0.05, ** *p* < 0.01, *** *p* < 0.001.

**Table 4:** L SMeans and Standard Errors of DSCC ranges according to M-5_DSCC_ model for milk components and groups of fatty acids (FA), and their orthogonal contrasts (*F*-value and significance).

Trait	DSCC ^1^ (%), LS Means				Contrasts, *F*-value and Significance		
	<63.5 (Class 1)	63.5–68.5 (Class 2)	68.6–73.5 (Class 3)	>73.5 (Class 4)	Class 2 + 3 DSCC	Class 2 DSCC	Class 2 + 3 DSCC
					vs.	vs.	vs.
					Low DSCC	Class 3 DSCC	High DSCC
N obs.	3041	392	403	909			
Milk components, %							
Fat	4.09 ± 0.19	4.03 ± 0.19	3.93 ± 0.19	3.81 ± 0.19	7.5 **	2.9	13.3 ***
Protein	3.49 ± 0.09	3.50 ± 0.09	3.52 ± 0.09	3.49 ± 0.09	2.6	1.7	2.6
Casein	2.74 ± 0.07	2.75 ± 0.07	2.76 ± 0.07	2.74 ± 0.07	2.1	1.4	2.8
Casein index ^2^	78.4 ± 0.14	78.4 ± 0.14	78.4 ± 0.14	78.4 ± 0.14	0.1	1.0	0.2
Lactose	4.83 ± 0.01	4.84 ± 0.01	4.85 ± 0.01	4.86 ± 0.01	9.0 **	3.5	4.6 *
Milk FA ^3^, g/100 g total FA							
SFA	68.5 ± 1.66	68.3 ± 1.66	68.1 ± 1.66	68.6 ± 1.66	3.1	0.7	3.5
UFA	30.7 ± 1.51	30.7 ± 1.52	31.3 ± 1.52	30.7 ± 1.52	2.8	5.0 *	2.0
MUFA	26.9 ± 1.36	27.1 ± 1.37	27.2 ± 1.37	26.7 ± 1.37	3.5	0.2	5.6 *
PUFA	4.64 ± 0.32	4.61 ± 0.32	4.69 ± 0.32	4.70 ± 0.32	0.2	3.9 *	3.1

^1^ DSCC = Differential Somatic Cell Count; ^2^ Casein index = casein to protein ratio, multiplied by 100; ^3^ FA = Fatty Acids; SFA = Saturated; UFA = Unsaturated; MUFA = Monounsaturated; PUFA = Polyunsaturated; * *p* < 0.05, ** *p* < 0.01, *** *p* < 0.001.

**Table 5:** LS Means and Standard Errors of DSCC ranges according to M-10_DSCC_ model for milk components and groups of fatty acids (FA), and their orthogonal contrasts (*F*-value and significance).

Trait	DSCC ^1^ (%), LS Means				Contrasts, *F*-Value and Significance		
	<58.5 (Class 1)	58.5-68.5 (Class 2)	68.6-78.5 (Class 3)	>78.5 (Class 4)	Class 2 + 3 DSCC	Class 2 DSCC	Class 2 + 3 DSCC
					vs.	vs.	vs.
					Low DSCC	Class 3 DSCC	High DSCC
N obs.	2609	824	759	553			
Milk components, %							
Fat	4.09 ± 0.19	4.05 ± 0.19	3.88 ± 0.19	3.82 ± 0.20	10.6 **	16.1 ***	4.9 *
Protein	3.49 ± 0.09	3.50 ± 0.09	3.51 ± 0.09	3.46 ± 0.09	2.4	2.4	6.6 *
Casein	2.74 ± 0.07	2.75 ± 0.07	2.76 ± 0.07	2.72 ± 0.07	1.9	1.8	5.9 *
Casein index ^2^	78.4 ± 0.14	78.4 ± 0.14	78.4 ± 0.14	78.4 ± 0.16	0.0	1.2	0.1
Lactose	4.82 ± 0.01	4.84 ± 0.01	4.86 ± 0.01	4.86 ± 0.01	28.4 ***	9.1 **	0.5
Milk FA ^3^, g/100 g total FA							
SFA	68.5 ± 1.66	68.4 ± 1.66	68.3 ± 1.66	68.8 ± 1.67	0.5	0.3	2.9
UFA	30.7 ± 1.51	30.8 ± 1.51	31.1 ± 1.51	30.4 ± 1.53	2.0	2.5	3.5
MUFA	26.9 ± 1.36	27.0 ± 1.36	27.0 ± 1.36	26.6 ± 1.38	0.6	0.0	3.0
PUFA	4.64 ± 0.32	4.62 ± 0.32	4.69 ± 0.32	4.65 ± 0.32	0.6	6.4 *	0.0

^1^ DSCC = Differential Somatic Cell Count; ^2^ Casein index = casein to protein ratio, multiplied by 100; ^3^ FA = Fatty Acids; SFA = Saturated; UFA = Unsaturated; MUFA = Monounsaturated; PUFA = Polyunsaturated; * *p* < 0.05, ** *p* < 0.01, *** *p* < 0.001.
